# Breaking barriers: enhancing cancer detection in younger patients by overcoming diagnostic bias in primary care

**DOI:** 10.3389/fmed.2024.1438402

**Published:** 2025-01-22

**Authors:** Waseem Jerjes, Daniel Harding

**Affiliations:** ^1^Research and Development Unit, Hammersmith and Fulham Primary Care Network, London, United Kingdom; ^2^Faculty of Medicine, Imperial College London, London, United Kingdom

**Keywords:** early-onset cancer, diagnostic bias, primary care, cancer detection, patient compliance

## Introduction

The issue of diagnostic bias within primary care has profound implications for the early detection of cancer, particularly among younger patients. Diagnostic bias refers to the preconceived notions and assumptions that influence a clinician's judgment, potentially leading to misdiagnosis or delayed diagnosis (1). Early diagnosis of cancer is critical for effective treatment and improved prognostic outcomes, making the recognition and mitigation of diagnostic biases an essential component of primary care practice.

Cancer incidence and mortality in adults under 50 years of age has been rising globally in the decades since 1990, especially in more highly-developed countries (2). This trend has occurred in the UK, and 9% of new cancer cases are diagnosed in those aged 25–49 (a rise in incidence of 22% from 1993 to 2019, total incidence 164.6 per 100,000 25–49 year olds in 2019), predominantly affecting women (Tables 1, 2) (3). Whilst this trend may partially be due to increasing access to investigations and improved diagnostic capabilities, it has been suggested that the rising incidence is also partly attributable to the oncogenic effects of rising obesity, rising alcohol consumption, and new dietary and environmental exposures (4).

**Table 1:** Cancer incidence rates per 100,000 population per year, subdivided by age group, in males and females aged 25**–**49 in the UK, from 2017**–**2019.

**Age**	**Male**	**Female**
**Cancer incidence rate per 100,000 population per year**		
25**–**29	47.1	70.3
30**–**34	67.0	119.7
35**–**39	90.7	177.4
40**–**44	126.9	268.6
45**–**49	215.4	418.0

**Table 2:** Percentages of different types of cancer as a proportion of the total incidence of new cancer diagnoses, in males and females aged 25–49 in the UK, from 2017–2019 (data from Cancer Research UK; N.B. all data excludes non-melanoma skin cancer) (3).

**Male**	**Female**
**Proportion of cancer incidence per type in 25–49 Year Olds**	
Testicular cancer 14%	Breast cancer 43%
Bowel cancer 11%	Melanoma 9%
Brain, central nervous system, or other intracranial cancer 10%	Cervical cancer 8%
Melanoma 10%	Thyroid 6%
Head and neck cancer 7%	Brain, central nervous system, or other intracranial cancer 6%
Other types of cancer 52%	Other types of cancer 32%

Primary care physicians are often the first point of contact for patients, positioning them uniquely to detect early signs of cancer. However, the age-related bias that younger patients are less likely to have serious conditions such as cancer can lead to significant delays in diagnosis (1, 5). This is especially concerning given that certain cancers in young patients can be more aggressive and progress rapidly, such as in breast cancer (6). Therefore, the main argument posited in this view point is that primary care physicians must actively work to overcome diagnostic biases that impede the early detection of cancer in younger patients.

### Diagnostic bias in younger patients

Diagnostic bias toward younger patients has the potential to lead to significant delays in identifying serious conditions such as cancer. Interviews with young adults with cancer, revealed that in many cases the patient and/or the clinician assumed it unlikely they would have cancer due to their age, resulting in delayed diagnosis in most cases (7). This bias is even noted in cancer investigation clinical guidance, with many guidelines having strict age cut-offs for investigating certain symptoms, such as under the UK's 2-week wait pathways (although some do contain overriding caveats for serious clinician concern) (8). Younger individuals presenting with atypical symptoms can be presumed by clinicians to have benign conditions (9), a presumption likely rooted in the significantly lower statistical prevalence of cancer within this demographic, (5, 10)something elsewhere termed epidemiological optimism bias (11). This bias is further exacerbated by the tendency of some primary care physicians to prioritize more common and less severe diagnoses (6). Studies have demonstrated that general practitioners are less likely to suspect malignancy in younger patients, which can result in delays in diagnosis and treatment (10, 12).

Research indicates that when younger patients present with symptoms such as unexplained weight loss, persistent pain, or unusual lumps, these signs are often attributed to benign causes like stress, infections, or minor injuries. For instance, a study by Dommett et al. (10) found that the likelihood of cancer being initially misdiagnosed in younger individuals was substantially higher compared to older adults. This misdiagnosis often leads to multiple consultations and a significant delay before appropriate investigations are conducted.

A notable example is the misdiagnosis of Hodgkin lymphoma in younger patients, which often presents with non-specific symptoms such as fatigue, fever, and lymphadenopathy. A delay in recognizing these symptoms as potential indicators of cancer can severely impact prognosis (10, 13). Similarly, younger patients with colorectal cancer often have a delay in diagnosis (14), due to both patient and doctor delay, and in some cases can also receive misdiagnosis as common benign conditions, such as hemorrhoids (15).

The tendency to overlook serious conditions in younger patients highlights the urgent need for heightened awareness and consideration of cancer as a differential diagnosis. Studies such as those by Lyratzopoulos et al. (16) have underscored the importance of considering a wider range of potential diagnoses to prevent delays that can compromise treatment outcomes. By recognizing and challenging these biases, we hope primary care physicians can improve diagnostic accuracy and ensure more timely intervention, ultimately enhancing patient care and survival rates.

### Importance of differential diagnosis and appropriate investigation

The necessity of considering cancer as a potential diagnosis in younger patients cannot be overstated. When cancer is not included as a differential diagnosis, critical time may be lost, leading to more advanced disease stages at the time of diagnosis. This delay can diminish the efficacy of treatment and worsens patient prognosis, making early and accurate diagnosis paramount (10, 11, 17). For example, a study by Swann et al. (18) reflects that delayed cancer diagnosis in younger patients often results in more aggressive disease progression and reduced survival rates. Conducted as a clinical audit in English general practices, data was collected on 17,042 patients newly diagnosed with cancer in 2014, noting that diagnostic delays occurred in 22% of cases due to patient, clinician, or system factors.

The thoroughness of the diagnostic process is crucial in mitigating these delays. Primary care physicians must adopt a comprehensive and systematic approach to evaluating symptoms, irrespective of the patient's age. This involves maintaining a high index of suspicion and conducting appropriate investigations even when initial symptoms are non-specific. As noted by Black et al. (19), implementing a structured diagnostic protocol can enhance early detection rates and improve clinical outcomes.

Vigilance in identifying potential cancer signs is imperative. Whilst of course the majority of presentations in primary care are not due to cancer, we would propose a dual approach of working toward a most likely diagnosis, whilst also considering serious differential diagnoses. For instance, non-specific symptoms such as abdominal pain, or unexplained weight loss, should prompt consideration of malignancy and inclusion of appropriate investigations regardless of patient age and whether malignancy is the most likely diagnosis. The integration of decision support tools and evidence-based guidelines in primary care practice can aid clinicians in making more informed diagnostic decisions (20, 21, 27). Additionally, a proactive stance, as recommended by O'Sullivan et al. (22), involves routine updates to clinical guidelines and continuous professional development to keep abreast of emerging trends in cancer presentation and investigation.

Beyond challenging diagnostic biases to improve clinician recognition of potential cancers in younger patients, there must be better support for clinicians in then making further decisions about investigations. An investigation can be judged in terms of its appropriateness, which is the balance of risk and benefit of any investigation for a specific patient (23). Unfortunately, there is generally a lack of research into the appropriateness of investigations specifically for cancer in younger people presenting to primary care (24). Changing this is vital to better inform guidelines and decision support tools, so that clinicians are well supported in making decisions about the investigations needed to diagnose cancer.

Apart from the potential benefit of an accurate diagnosis, there are innumerable risks of an investigation a clinician will be weighing up, such as: false reassurance and patient disengagement following a false negative result; patient anxiety and further investigations resulting from either false positive results or non-symptomatic incidental findings; and the risks of direct harm from any investigation, for example radiation exposure. More broadly, clinicians will also be considering the wider implications of any investigation, including the costs to the healthcare system and the risk of lengthening waiting times for investigations for other patients. These risks are especially pronounced in younger patients presenting to primary care, as the vast majority of presentations, will not be due to cancer. To make more confident decisions about investigations, clinicians must be given enhanced guidelines for managing investigations for potential cancer in younger people, as well as better data to back-up their decisions. This research and guidance should also account for possible lead-time bias, which would be the potential for any increase in investigation in younger people for cancer, to lead to earlier detection but not necessarily truly enhanced survival (25).

### Training and awareness programs

The implementation of comprehensive training and awareness programs for general practitioners (GPs) is critical in addressing diagnostic biases and improving the accuracy of cancer detection in younger patients. Such programs are designed to enhance clinicians' awareness of their biases, be they conscious or unconscious, and equip them with the necessary skills to recognize atypical presentations of cancer. These educational initiatives can significantly alter diagnostic practices and improve patient outcomes (Table 3). An excellent example of this is Bowel Cancer UK's “Never Too Young” initiative (26).

**Table 3:** Summary of key strategies to mitigate diagnostic bias in cancer detection among younger patients.

**Strategy**	**Description**	**Expected outcome**
Comprehensive diagnostic protocols	Implementation of structured diagnostic protocols based on evidence, including age-specific guidelines to reduce age-related biases in diagnosis.	Improved early detection rates, diagnostic accuracy, and appropriateness of diagnostic procedures.
Training and awareness programs	Education and training to enhance clinicians' awareness of biases and equip them with skills to recognize atypical cancer presentations. This includes case-based learning and simulation exercises.	Increased clinician awareness, reduced diagnostic errors, and improved management of atypical cases.
Patient-centered care	Extended consultation times, shared decision-making, improved communication channels, and greater patient engagement in their diagnostic journey.	Enhanced patient satisfaction, trust, and engagement, leading to more accurate and timely diagnoses.
Decision support tools	Integration of AI and other decision support tools to provide real-time diagnostic guidance, with recommendations grounded in solid evidence of net clinical benefit.	Reduction in diagnostic errors, improved decision-making processes, and safer diagnostic practices.
Multidisciplinary teams	Collaboration with specialists such as oncologists, radiologists, and pathologists to provide a more comprehensive and evidence-based diagnostic approach.	Reduced diagnostic errors, holistic evaluation of potential cancer diagnoses, and improved outcomes.
Empathetic communication	Training in empathetic communication to build stronger patient-provider relationships, with emphasis on active listening and validating patient concerns.	Increased patient trust, adherence to diagnostic procedures, and improved diagnostic outcomes.
Diagnostic appropriateness tools	Utilization of resources such as the ACR Appropriateness Criteria, NICE Guidelines, and similar evidence-based tools to ensure diagnostics align with clinical indications.	Reduced overdiagnosis and overtreatment, optimal allocation of healthcare resources, and fewer delays.
Reflective practice and audits	Regular audits of diagnostic practices and reflective exercises to evaluate and address potential biases in clinicians' diagnostic approaches.	Continuous improvement in diagnostic quality and adherence to best practices.

Effective training programs focus on several key areas. Firstly, they emphasize the importance of a thorough and systematic approach to diagnosis, encouraging GPs to consider a broad differential diagnosis that includes malignancies, regardless of the patient's age (19). According to a study by Walter et al. (28), training that incorporates case-based learning and simulation exercises can improve diagnostic accuracy by providing GPs with practical experience in identifying cancer symptoms in younger patients. These programs also highlight the significance of early detection and the potential consequences of delayed diagnosis, reinforcing the need for vigilance and overcoming diagnostic bias in clinical practice.

Furthermore, awareness initiatives that target diagnostic bias can help clinicians recognize and mitigate their own preconceived notions. A study by Staal et al. (29) examined the importance of fostering a broad differential diagnostic approach, as narrowing the focus prematurely can overlook potential key diagnoses. Incorporating reflective practice, where physicians regularly review and critically analyze their diagnostic decisions, can foster a culture of self-awareness, openness and continuous improvement.

In addition to formal training, ongoing professional development and access to updated clinical guidelines are essential. Regular workshops, educational courses, and peer-reviewed journals help keep GPs up to date with the latest evidence-based practices and emerging trends in cancer diagnosis. For instance, the integration of decision support tools, as recommended by Schmidt et al. (30), can assist GPs in making more informed decisions by providing contemporary guidance based on current clinical data.

## Discussion

The necessity to overcome diagnostic biases in primary care to enhance early cancer detection in younger patients has been examined in the preceding sections. The central points underscored the detrimental impact of age-related biases, the importance of including cancer in differential diagnoses for younger patients, and the vital role of training and awareness programs (1, 10). Addressing these biases has significant implications for clinical practice, patient outcomes, and the broader healthcare system.

One of the primary implications for practice is the potential improvement in early cancer detection rates among younger patients (17). Early diagnosis is crucial as it often leads to better prognostic outcomes and more effective treatment options (5). By ensuring that cancer is considered as a possible diagnosis irrespective of patient age, primary care physicians can help mitigate the risks associated with delayed diagnosis (19, 21). Moreover, addressing diagnostic biases can enhance the overall quality of patient care. When physicians adopt a more inclusive diagnostic process, they are likely to conduct more thorough evaluations, thereby improving the accuracy of their diagnoses (20, 22). This comprehensive approach not only benefits the patients by providing timely and appropriate care but also reinforces trust in the healthcare system. To actively engage primary care physicians in mitigating diagnostic biases, ongoing education and awareness-raising must be emphasized. Professional development programs that focus on recognizing and overcoming diagnostic biases should be mandated (22).

Furthermore, implementing decision support algorithms in primary care settings can significantly aid in reducing diagnostic errors. Algorithms can provide evidence-based guidance and highlight potential malignancies based on presenting symptoms, regardless of patient age. The use of artificial intelligence (AI) in primary care is one area that may see future expansion and work alongside this. AI tools can analyze large datasets to identify patterns that may not be immediately apparent to human clinicians, which could offer enhanced diagnostics and in the future (31). However, this is dependent on the specifics of any AI development and implementation, and there are concerns being raised of AI amplifying and entrenching the existing human diagnostic biases of those designing and developing it (32, 33).

Primary care settings should also advocate for policy changes that support regular training and the integration of diagnostic support tools. Policymakers and healthcare administrators need to allocate resources (both in terms of finances and clinician time) toward these initiatives, recognizing their long-term benefits in improving patient outcomes and reducing healthcare costs associated with late-stage cancer treatments (28), alongside the important moral imperative to challenge factors that disadvantage younger people with cancer. Collaborations with academic institutions, professional organizations, and charities, can help facilitate the development and dissemination of effective training and policy changes (26, 27, 34, 35).

Future research should focus on several key areas of cancer diagnosis in younger people. Firstly, it is vital research looks to further develop and validate diagnostic algorithms that are tailored to younger patient populations, so that clinicians can be better supported in their decision making. Studies should also investigate the effectiveness of different training methodologies in reducing diagnostic biases and improving early detection rates (13, 18, 19, 36, 37). Emerging population-based evidence supports this trend as particularly urgent. Sifaki-Pistolla et al. (38) demonstrated a significant increase in the incidence of colorectal cancer among age groups below 50 years during the past 30 years in the Greek population, while further projection indicated that there is also a trend to be projected. These findings set the challenge for updating the guidelines by putting an accent on young age groups early in the course of interventions.

Additionally, research on patient outcomes following the implementation of bias reduction strategies can provide valuable insights into the practical benefits of these initiatives. There is a broader picture here as well, which has innumerable areas where future research would be helpful, including research looking to understand and intervene in patient factors related to delayed presentation in younger people with cancer, as well as research examining the underlying reasons for the rising incidence of cancer diagnosis in younger people. Besides, cultural and behavioral factors play an important role in the influence of delays in diagnosis. Oikonomidou et al. indicated that in rural Greece, patients often refused diagnostic procedures such as endoscopy due to fears, misconceptions, and competing life priorities. These barriers underline the need for culturally sensitive strategies to enhance compliance and early detection (39).

### Patient-centered care strategies

Addressing diagnostic biases in primary care not only requires systemic and educational interventions but also necessitates a shift toward more patient-centered care strategies. These strategies place the patient at the heart of the diagnostic process, ensuring that their concerns and symptoms are thoroughly evaluated and addressed. One effective patient-centered strategy is the implementation of extended consultation times for complex cases, allowing GPs to conduct more comprehensive histories and examinations, and consider a wider range of differential diagnoses. Research by Epstein et al. (34) suggests that longer consultation times are associated with improved diagnostic accuracy, particularly in cases presenting with atypical symptoms.

Another crucial aspect of patient-centered care is the active involvement of patients in their diagnostic journey. This can be achieved through shared decision-making, where patients are encouraged to participate more actively in discussions about their symptoms, investigations, and differential diagnoses. Providing patients with detailed information about their symptoms can empower them to advocate for their own health. A study by Charles et al. (35) found that patient involvement in the diagnostic process leads to higher satisfaction and better health outcomes. Concurrently, improving communication channels between GPs and patients is essential. Timely follow-ups and open lines of communication can help in monitoring the progression of symptoms and making appropriate adjustments to the diagnostic approach. Implementing electronic health records (EHR) with patient portals can facilitate this communication.

Patient education is a critical public health and policy component of patient-centered care. Educating patients about the signs and symptoms of cancer, regardless of their age, can raise awareness and prompt earlier medical consultations. Community outreach programs and educational campaigns, as highlighted by Young and Robb (36) have been shown to improve public awareness of cancer symptoms, which can lead to more timely presentation and diagnosis.

Integrating multidisciplinary teams into primary care can also enhance patient-centered care. Collaboration with specialists such as oncologists, radiologists, and pathologists can provide a more comprehensive approach to diagnosis and treatment planning (35–37).

Lastly, fostering a supportive and empathetic clinical environment is paramount. Primary care physicians should be trained in empathetic communication, which involves actively listening to patients, validating their concerns, and expressing genuine care and understanding. Empathetic interactions have been shown to build stronger patient-provider relationships, increase patient trust, and improve adherence to recommended diagnostic procedures (37).

## Conclusion

Addressing diagnostic biases in primary care is paramount for improving the detection of cancer in younger patients. Overcoming these biases requires a multifaceted approach, including comprehensive training programs to enhance clinical awareness, the integration of decision support tools, and systemic changes to support continuous professional development. Furthermore, adopting patient-centered care strategies, such as extended consultation times, shared decision-making, and improved communication, can significantly enhance diagnostic accuracy and patient outcomes. By challenging diagnostic biases, and fostering an environment of vigilance and empathy, we hope primary care physicians can better identify and diagnose cancer in younger patients, ultimately leading to more timely and effective treatments. This proactive and inclusive approach not only benefits patients but also wider healthcare systems.
